# Differences in health related quality of life between men and women with chronic pain: results from a nationwide cross-sectional survey in the general Spanish population

**DOI:** 10.1186/s12889-026-26688-8

**Published:** 2026-03-12

**Authors:** Helena De Sola, María Dueñas, Alejandro Salazar, Ana Esquivias, Sara Rubio, Inmaculada Failde

**Affiliations:** 1 Department of General Economics, Area of Sociology, University of Cádiz, Puerto Real, 11510 Spain; 2 Observatory of Pain, Grünenthal Foundation-University of Cadiz, Cádiz, 11009 Spain; 3 Biomedical Research and Innovation Institute of Cadiz (INiBICA), Cádiz, 11009 Spain; 4 Department of Statistics and Operational Research, University of Cádiz, Puerto Real, 11510 Spain; 5 Medical Department, Grünenthal Pharma, S.A., Madrid, Spain; 6 Market Access Department, Grünenthal Pharma, S.A, Madrid, Spain; 7 Preventive Medicine and Public Health Area, University of Cádiz, Cádiz, 11009 Spain

**Keywords:** Chronic pain, Sex/gender, Mental health, Physical health, Health related quality of life

## Abstract

**Background:**

Taking into account the differences between sexes/genders in the chronic pain (CP) process and the influence of the biopsychosocial factors in the health-related quality of life (HRQL), this study aims to determine the differences between women and men with CP and the factors associated with their mental and physical HRQL.

**Methods:**

A nationwide cross-sectional study was carried out in 7,058 participants representative of the Spanish adult population. Sociodemographic variables, presence and characteristics of CP, daily living limitations, sleep problems, anxiety and depression (HADS), quality of life (SF-12v2), social support (DUKE-UNC-11) were collected. Four linear regression models were generated.

**Results:**

1,825 people had CP with an average 51.52 years old (SD:15.58), 58.7% being women. The variables associated with lower scores on physical component (PCS-12) were older age (women B=-0.196;men B=-0.151), higher pain intensity (women B=-1.557;men B=-1.435), higher depression (women B=-0.404;men B=-0.322), needed sick leave (women B=-3.816;men=-3.439), reported disability (women B=-10.541;men B=-7,544), retired (women B=-2.454;men B=-1.937), unemployed women (B=-3.394), fibromyalgia diagnostic (B=-4.787), men with arthrosis (B=-2.521), neuropathy (B=-3.871) and low back pain (B=-2.199). Men with university studies obtained higher scores on PCS-12 (B = 2.079). The variables associated with mental component (MCS-12) showed that younger people (women B = 0.111;men B = 0.058), higher anxiety (women B=-1.067;men B=-0.101), and depression (women B=-0.817;men B=-1.004), women with herniated disc (B=-5.324), and endometriosis (B=-3.975), scored lower on MCS-12. Women with secondary studies (B = 1.699), men with pain located in neck (B = 3.785), back (B = 3.108), and joints (B = 4.375) and men with higher social support (B = 0.073) obtained higher score on MCS-12.

**Conclusions:**

CP affects HRQL among both sexes. However, differences exist depending on the variables. Identifying subjects with these factors is fundamental to preventing their HRQL from worsening.

## Background

 Chronic pain (CP) has been considered an illness itself [1] and it is a health problem that has reached pandemic proportions [2, 3]. The prevalence of CP ranges from 10% to 30% in Europe [4, 5] and around 25.9% in Spain [6].

Several studies have been focused on sex and gender differences in CP, showing that women generally experience more pain across their lifespan compared with men [7, 8]. Experimental research in laboratory environments shows that women are more sensitive to pain, report higher pain intensity, use more analgesic medication, and more often report widespread pain. Likewise, some studies have examined biological differences in response to the treatment of chronic pain [9], and how pain influences psychological aspects, making women more directly affected [10]. These characteristics of pain could be explained by neurological and genetic factors, but also by hormonal factors, which can act as sex-specific pain mediators.

Additionally, there are studies that take gender role influence into account in the perception of pain and the way it is reported. Boys and men are taught to be tough, tolerate pain and endure painful experiences, while girls and women are socialized to be sensitive, careful and to verbalize discomfort [11]. Additionally, the sex/gender differences related to work and family tasks have been analyzed, showing them to have an impact on health and on the painful disease, women being worse affected since they are exposed to more precarious tasks and conditions [12–14].

The need to include both sex and gender in pain research has been argued critically [11]. It is difficult to dissociate sex and gender since both influence the experience of pain. Thus, the term sex/gender used by the authors of this study has started to be used, highlighting the continuing difficulty in disassociating them, and the need to consider them interacting factors [15].

The experiences of people living with CP have been widely studied because of their impact on daily life and their quality of life. The health-related quality of life (HRQL) of people with CP has been shown to be worse than that of the general population, usually related to physical limitations and disability conditions, as well as their impaired mental well-being. Previous studies have investigated whether HRQL differs in women and men, reporting that women’s HRQL is almost always lower than men’s [16, 17]. HRQL is a multidimensional and complex construct that is determined by a set of factors that differ depending on sex/gender such as biological, sociodemographic (marital status, professional status.), and psychological factors [18, 19]. For this reason, it is important to gain a better understanding of the differences in HRQL in people with CP according to sex/gender. Furthermore, despite the high prevalence of CP in Spain, which has been reported to be increasing (from 16.6% to 25.9% in recent years) [6, 20], and the significant sex/gender disparity observed [21], a knowledge gap persists regarding the characteristics, consequences, and factors between women and men in this country.

Taking into account the differences between sexes/genders in the CP process and the influence of the biopsychosocial factors in the HRQL, this study aims to determine the sociodemographic differences, characteristics and consequences of the pain, state of health, and the use of the health system among women and men with CP in the Spanish population and the factors associated with their HRQL.

## Methods

### Study design

This study is a secondary analysis of the data collected in a cross-sectional study called “Barometro del Dolor” (Pain Barometer) carried out on a representative sample of the general Spanish population (18–85 years). The data were obtained to determine the prevalence and characteristics of CP in the Spanish population.

### Study population

The original study included 7,058 subjects. Here, we restricted the population to those with CP, a subsample of 1,825 participants (1,072 women and 753 men).

### Sampling method

The study population was obtained through volunteer sampling from a census property of the company CINT (platform for gathering digital information oriented exclusively to purposes related with market research, providing access to panels of participants that comply with the requirements of the European Society for Opinion and Marketing Research, ESOMAR and the International Organization for Standardization, ISO) collected by “*More than Research*”, a Spanish market research agency [22].

For the aim of the original study [6] a sample size was calculated to estimate a prevalence of CP of 28.4% (estimated from preliminary results from a pilot study in the target population), with a 95% confidence level and accuracy (or margin of error) of 13%. The term accuracy indicates how close the sample estimate is expected to be to the true population value. To guarantee representativeness by sex, age and Autonomous Community (specific territorial boundaries in Spain), quotas by strata were established proportionally to the general population distribution. For the objective of this study, taking into account our sample of 1,825 (1,072 women and 753 men) and the data obtained in Casals et al. in the scores of MCS and PCS of the SF-12 in men and women with CP [23], with a 95% confidence level, it has been estimated a power of 48% for the mean difference in MCS and a power of almost 100% for PCS.

Data was collected using a mixed-mode approach. For individuals aged 18 to 75, we used Computer-Assisted Web Interviewing (CAWI), a self-administered data collection method where respondents complete a questionnaire online via an electronic device. For the older demographic (individuals aged 76 to 85), we used Computer-Assisted Telephone Interviewing (CATI), where participants are asked via phone call. A total of 7,058 interviews were obtained (6,394 CAWI and 664 CATI).The study was performed in line with the principles of the Declaration of Helsinki. Participants are members of a panel that complies with the requirements of the ESOMAR, and are already doubly committed to participating in market research studies. All interviewees provided explicit informed consent at the start of the questionnaire. While initial contact (via CATI or CAWI platform) meant the data collectors were aware of participants’ identities, all data were processed using pseudonymization. This means that personal identifiers were immediately stripped from the survey responses and stored separately. In this way, the company that conducted the interview provided us the data anonymized. This process guarantees the confidentiality and privacy of the information obtained, as no personal information was linked to the final analysis dataset, in accordance with all ethical standards.

### Data collection and measures

Data were collected between June 13th and June 20th, 2022. Two screening questions were used to identify the people with CP in accordance with the criteria of the International Association for the Study of Pain (IASP), and an individual was considered to have CP if the frequency of their pain was at least 4 or 5 days a week during the previous month and if their pain had lasted for three or more months. They were considered to have CP if they answered “yes” to both questions. The data collected included: sociodemographic data (age, gender/sex and educational level, employment status); the characteristics of the pain (intensity, duration and location of the painful sites and diagnoses). Pain intensity over the past week was measured using a 0–10 numerical rating scale, where 10 is the worst imaginable pain. The limitations to activities of daily living due to pain referred to the four previous week were also collected (feeding, sitting, getting up from a chair or bed, lying down, dressing and undressing, going to the bathroom, showering or bathing). The response options provided for each activity were: “Yes, a lot,” “Yes, somewhat,” or “No, I have not limited them.” Additionally, Sleep problems due to pain (yes/no), presence and level of anxiety and depression, quality of life and social support were also collected. Healthcare information was also collected (number of medical consultations and specialty where had been attended in the previous year and length of time on the waiting list).

Anxiety and depression were measured with the Hospital Anxiety and Depression Scale (HADS) [24]. It consists of 14 items grouped in two subscales (one for anxiety and one for depression), each with seven items. Each subscale is scored from 0 to 21, where higher scores denote higher levels of anxiety/depression. Scores over 10 indicate the presence of these pathologies, and scores between 8 and 10 are considered to be doubtful cases of anxiety or depression [25]. Internal consistency for the sample was satisfactory, with Cronbach’s alpha values of 0.856 for anxiety and 0.860 for depression.

To evaluate health related quality of life, the 12-item Short-Form Health Survey version 2 (SF-12v2) was used. This tool includes 12 elements that make up its profile of eight dimensions: physical functioning, role physical, bodily pain, general health, vitality, social functioning, role emotional and mental health. It also includes two global ratings: the PCS-12 for physical health and the MCS-12 for mental health. The scores for these two summary dimensions range from 0 to 100 with the highest scores indicating a better quality of life [26]. Reliability analysis for our sample indicated that both the PCS-12 and MCS-12 subscales exhibited acceptable internal consistency (alpha = 0.812 and alpha = 0.756, respectively). For this analysis, only the PCS and MCS scores were included, which were standardized with a median of 50.

Perceived social support was measured using the Duke-UNC-11 functional social support questionnaire [27]. It consists of 11 items grouped into two subscales: confidential support (7 items) and affective support (4 items). The former represents the ability to communicate with other people, and the latter evaluates the affection and empathy received, as well as the readiness of people to welcome or meet with them. It has a global score ranging from 11 to 55, where higher scores indicate higher perceived social support. Scores below 32 are considered a sign of low perceived social support. In our sample, the scale showed a good reliability with a Cronbach’s alpha of 0.94.

### Statistical analysis

The differences between women and men were evaluated with bivariate analyses. The χ2 test was used for categorical variables and a Mann-Whitney U test for continuous variables with a non-normal distribution. Variable distribution was tested using the Kolmogorov-Smirnov test. No correction for multiple testing was applied, as the bivariate analyses were designed to assess distinct pairwise associations rather than to test a unified hypothesis. Each comparison was considered an independent exploratory analysis intended to provide descriptive insight rather than to yield jointly interpretable inferential conclusions. A significance level α = 0.05 was established.

Initially, to address the second objective (analysing the factors associated with HRQOL) two multiple regression models were constructed in the whole sample. The PCS-12 and MCS-12 scores served as the dependent variables, with gender and other variables included as covariates. Interactions of gender with other covariates were also evaluated to determine differential effects. However, the results in both models showed no statistical significance when including gender as a covariate or with the addition of interaction terms. On the other hand, given our research question, we were also interested in allowing the set of covariates potentially associated with HRQOL to differ between men and women. Consequently, we constructed four separate multiple linear regression models to analyse the factors associated with HRQOL:

- The physical component PCS-12 of women with CP (Model 1).
- The physical component PCS-12 of men with CP (Model 2).
- The mental component MCS-12 of women with CP (Model 3).
- The mental component MCS-12 of men with CP (Model 4).

Targeting the mental and physical components as the dependent variable. The criteria used to select the covariates included in these models were both statistical (a significant difference observed in the bivariate analysis: *p* < 0.05) and clinical (previously shown in the literature [21, 28–30]). In addition, to facilitate the interpretation of the model, the categories of the variables related to the diagnosis were dichotomised as ‘yes’ or ‘no’.

The stepwise method was used for variable selection in all models. Given the number of potential confounders and exposure variables available, this approach allowed for a systematic search for the most parsimonious and statistically significant combination of predictors, without relying solely on a priori subjective criteria. The Wald test was used to test the significance of the parameters of each covariate, along with its clinical relevance. The adjusted R^2^ was considered as the goodness-of-fit measure in the case of the multiple linear regression. In the multiple linear regression model, tolerance and the variance inflation factor (VIF) were computed. We assumed that collinearity was not present when the VIF value was below 5 and the tolerance score over 0.2. Furthermore, the assumption of normality of residuals was assessed using the Shapiro-Wilk test, the assumption of homoscedasticity was evaluated by the Breusch-Pagan test, and the assumption of independence of errors was tested using the Durbin-Watson statistic (values near 2 indicate independent residuals).

All assumptions were met for the four multiple linear regression models (data not shown).

All the analyses were carried out with IBM SPSS V29, and the plots with Excel 365.

## Results

### General characteristics of people with CP

The population with CP was composed of 1,825 people and their average age was 51.52 years (SD = 15.58). This population included 1,072 women (58.7%) with a mean age of 51.51 years (SD = 16.14) (Table 1).

**Table 1:** Sociodemographic characteristics of people with chronic pain and differences by sex

Variables	Categories	Global (N=1825)		Men (N=753)		Women (N=1072)		p
		n	%	n	%	n	%	
Sociodemographic data								
Age	Mean (SD)	51.52 (15.58)		51.54 (14.77)		51.51 (16.14)		0.971^a^
	Median (IR)	5 (2)		51 (21)		52 (24)		
Age	18-34	300	16.4	116	15.4	184	17.2	0,231^b^
	35-54	719	39.4	316	42	403	37.6	
	55-75	639	35	259	34.4	380	35.4	
	76-85	167	9.2	62	8.2	105	9.8	
Autonomous Community	Andalusia	323	17.7	155	20.6	168	15.7	0,049^b^
	Aragón	54	3	28	3.7	26	2.4	
	Asturias	39	2.1	12	1.6	27	2.5	
	Balearic Islands	50	2.7	21	2.8	29	2.7	
	Canary Islands	84	4.6	37	4.9	47	4.4	
	Cantabria	21	1.2	5	0.7	16	1.5	
	Castile and León	84	4.6	34	4.5	50	4.7	
	Castile - La Mancha	64	3.5	26	3.5	38	3.5	
	Catalonia	316	17.3	107	14.2	209	19.5	
	Community of Valencia	199	10.9	84	11.2	115	10.7	
	Extremadura	51	2.8	15	2	36	3.4	
	Galicia	113	6.2	45	6	68	6.3	
	Madrid	258	14.1	115	15.3	143	13.3	
	Murcia	55	3	22	2.9	33	3.1	
	La Rioja	10	0.5	3	0.4	7	0.7	
	Navarra	21	1.2	11	1.5	10	0.9	
	Basque Country	83	4.5	33	4.4	50	4.7	
Educational level	No eduction	19	1	6	0.8	13	1.2	0,202^b^
	Primary education	134	7.3	47	6.2	87	8.1	
	Secondary education	401	22	185	24.6	216	20.1	
	Vocational Training	535	29.3	219	29.1	316	29.5	
	University education	730	40	294	39	436	40.7	
	No response	6	0.3	2	0.3	4	0.4	
Employment status	Currently in work	921	50.5	426	56.6	495	46.2	<0.001^b^
	I am unemployed	198	10.8	74	9.8	124	11.6	
	I am retired	429	23.5	192	25.5	237	22.1	
	Absolute permanent validity	107	5.9	43	5.7	64	6	
	I am studying	52	2.8	9	1.2	43	4	
	Unpaid housework	118	6.5	9	1.2	109	10.2	
Sick leave (Only those currently	Yes	428	46.5	194	45.5	234	47.3	0,599^b^
working answer)	No	493	53.5	232	54.5	261	52.7	

*SD* Standard deviation, *IR* Interquartile range

^a^Mann-Whitney U

^b^Chi-squared

### Differences between women and men with CP

One of the main differences between men and women is their occupational status; most of the men (56.6%) reported to be working at the time of the interview and the percentage of domestic work was higher in women (10.2%). Furthermore, significant variation was noted across the Autonomous Communities. Specifically, the highest prevalences were registered among men in Andalusia (20.6%) and Madrid (15.3%), and among women in Catalonia (19.5%) (Table 1).

Regarding health status, compared to the men, the women presented the following results: lower scores for the mental component on the SF-12 scale (MCS-12) (41.27 SD = 11.56 vs. 44.07 SD = 11.53), more cases of anxiety (31.8% vs. 21.6%), higher scores for level of anxiety (8.45 SD = 4.57 vs. 7.43 SD = 4.3) and more cases of depression (24.3% vs. 19.1%) (Table 2).

**Table 2:** Clinical characteristics of people with chronic pain and differences by sex

Variables	Categories		Global (N=1825)		Men (N=753)		Women (N=1072)		p
			n	%	n	%	n	%	
**HEALTH STATUS**									
Quality of life (SF 12v.2) PCS	Mean (SD) Median (IR)		37.98 (10.5) 38.71 (15.65)		38.17 (10.09) 39.12 (14.87)		37.84 (10.78) 38.2 (16.5)		0.483^a^
Quality of life (SF 12v.2) MCS	Mean (SD) Median (IR)		42.43 (11.62) 41.85 (16.5)		44.07 (11.53) 43.71 (15.92)		41.27 (11.56) 40.39 (16.43)		<0.001^a^
HADS- Anxiety	Mean (SD) Median (IR)		8.03 (4.49) 8 (6)		7.43 (4.3) 7 (6)		8.45 (4.57) 8 (7)		<0.001^a^
HADS- Anxiety	No case Doubtful case Case		857 464 504	47 25.4 27.6	395 195 163	52.5 25.9 21.6	462 269 341	43.1 25.1 31.8	<0.001^b^
HADS- Depression	Mean (SD) Median (IR)		7.21 (4.46) 7 (6)		7.07 (4.34) 7 (5)		7.31 (4.53) 7 (6)		0.226^a^
HADS- Depression	No case Doubtful case Case		1005 415 405	55.1 22.7 22.2	431 178 144	57.2 23.6 19.1	574 237 261	53.5 22.1 24.3	0,030^b^
DUKE TOTAL	Mean (SD) Median (IR)		37.12 (11.55) 38 (16)		36.75 (11.16) 37 (15)		37.38 (11.82) 38 (17)		0.127^a^
DUKE CATEGORICAL	Low Normal		529 1296	29 71	212 541	28.2 71.8	317 755	29.6 70.4	0,511^b^
**PAIN**									
Duration of chronic pain in months	Mean (SD) Median (IR)		81.91 (100.91) 48 (96)		80.74 (103.66) 38 (96)		82.75 (98.95) 48 (96)		0.277^a^
Diagnosed cause of CP*(Possible more than one)*	Arthrosis	Yes No	603 1222	33 67	221 532	29.3 70.7	382 690	35.6 64.4	0,005^b^
	Osteoporosis	Yes No	600 1225	32.9 67.1	219 534	29.1 70.9	381 691	35.5 64.5	0,004^b^
	Neck pain	Yes No	843 982	46.2 53.8	318 435	42.2 57.8	525 547	49 51	0,004^b^
	Lumbar pain	Yes No	1061 764	58.1 41.9	445 308	59.1 40.9	616 456	57.5 42.5	0,486^b^
	Trauma	Yes No	264 1561	14.5 85.5	122 631	16.2 83.8	142 930	13.2 86.8	0,077^b^
	Migraine or other chronic headaches	Yes No	574 1251	31.5 68.5	173 580	23 77	401 671	37.4 62.6	<0.001^b^
	Pain related to surgery	Yes No	275 1550	15.1 84.9	133 620	17.7 82.3	142 930	13.2 86.8	0,009^b^
	Rheumatoid arthritis	Yes No	330 1495	18.1 81.9	137 616	18.2 81.8	193 879	18 82	0,917^b^
	Sciatica	Yes No	468 1357	25.6 74.4	197 556	26.2 73.8	271 801	25.3 74.7	0,671^b^
	Muscles contractures	Yes No	923 902	50.6 49.4	353 400	46.9 53.1	570 502	53.2 46.8	0,008^b^
	Cancer	Yes No	66 1759	3.6 96.4	21 732	2.8 97.2	45 1027	4.2 95.8	0,112^b^
	Shoulder	Yes No	533 1292	29.2 70.8	215 538	28.6 71.4	318 754	29.7 70.3	0,607^b^
	Fibromyalgia	Yes No	190 1635	10.4 89.6	38 715	5 95	152 920	14.2 85.8	<0.001^b^
	Diabetic neuropathy or other neuropathy	Yes No	140 1685	7.7 92.3	61 692	8.1 91.9	79 993	7.4 92.6	0,563^b^
	Carpal tunnel syndrome	Yes No	223 1602	12.2 87.8	81 672	10.8 89.2	142 930	13.2 86.8	0,110^b^
	Crohn’s disease or ulcerative colitis	Yes No	74 1751	4.1 95.9	28 725	3.7 96.3	46 1026	4.3 95.7	0,542^b^
	Herniated disc	Yes No	21 1804	1.2 98.8	9 744	1.2 98.8	12 1060	1.1 98.9	0,881^b^
	Cause of pain unknown	Yes No	495 1330	27.1 72.9	191 562	25.4 74.6	304 768	28.4 71.6	0,157^b^
Where is the worst pain located	It is widespread pain Head Neck (cervical) Back Limbs and/or joints Chest Abdomen Other		240 165 193 511 603 18 73 20	13.2 9.1 10.6 28 33.1 1 4 1.1	67 61 82 251 248 10 25 8	8.9 8.1 10.9 33.4 3 1.3 3.3 1.1	173 104 111 260 355 8 48 12	16.2 9.7 10.4 24.3 33.1 0.7 4.5 1.1	<0.001^b^
Sleep disorder due to CP	Yes No		1182 643	64.8 35.2	467 286	62 38	715 357	66.7 33.3	0,039^b^
Pain intensity	Mean (SD) Median (IR)		6.78 (1.72) 7 (2)		6.6 (1.71) 7 (2)		6.9 (1.71) 7 (2)		<0.001^a^
Pain intensity	Slight or very slight (0-3) Moderate (4-6) Severe (7-9) Unbearable (10)		85 608 1051 81	4.7 33.3 57.6 4.4	43 275 408 27	5.7 36.5 54.2 3.6	42 333 643 54	3.9 31.1 60 5	0,009^b^
**CONSEQUENCES CP**									
Limitations in daily activities due to CPFeeding oneself	Yes, many Yes, some No limitations		113 363 1349	6.2 19.9 73.9	34 126 593	4.5 16.7 78.8	79 237 756	7.4 22.1 70.5	<0.001^b^
Limitations in daily activities due to CPSitting down	Yes, many Yes, some No limitations		215 710 900	11.8 38.9 49.3	86 283 384	11.4 37.6 51	129 427 516	12 39.8 48.1	0,484^b^
Limitations in daily activities due to CPGetting up from chair or bed	Yes, many Yes, some No limitations		322 810 693	17.6 44.4 38	121 333 299	16.1 44.2 39.7	201 477 394	18.8 44.5 36.8	0,243^b^
Limitations in daily activities due to CPLying down	Yes, many Yes, some No limitations		234 666 925	12.8 36.5 50.7	95 252 406	12.6 33.5 53.9	139 414 519	13 38.6 48.4	0,052^b^
Limitations in daily activities due to CPDressing and undressing	Yes, many Yes, some No limitations		171 699 955	9.4 38.3 52.3	67 315 371	8.9 41.8 49.3	104 384 584	9.7 35.8 54.5	0,034^b^
Limitations in daily activities due to CPGoing to the toilet	Yes, many Yes, some No limitations		138 395 1292	7.6 21.6 70.8	57 163 533	7.6 21.6 70.8	81 232 759	7.6 21.6 70.8	>0.999^b^
Limitations in daily activities due to CPShowering or bathing	Yes, many Yes, some No limitations		163 491 1171	8.9 26.9 64.2	66 201 486	8.8 26.7 64.5	97 290 685	9 27.1 63.9	0,956^b^
Sick leave due to pain in the last year	Yes No		474 1186	28.6 71.4	213 480	30.7 69.3	261 706	27 73	0,096^b^
Time on sick leave in last year (days)	Mean (SD) Median (IR)		136.74 (130.37) 90 (180)		146.09 (131.34) 90 (210)		129.14 (129.33) 90 (180)		0.056^a^
Stopped or changed work duties due to CP(Only those answering “yes” to previous question answer)	Yes, I had to stop Yes, I changed duties No		153 82 239	32.3 17.3 50.4	72 42 99	33.8 19.7 46.5	81 40 140	31 15.3 53.6	0,249^b^
**USE OF HEALTHCARE SYSTEM DUE TO CP**									
Received healthcare in the last four weeks	Yes No		769 1056	42.1 57.9	337 416	44.8 55.2	432 640	40.3 59.7	0,058^b^
Frequency attending Primary Care(Only those answering “yes” to previous question answer)	Never Once Twice 3 or more times		102 408 168 91	13.3 53.1 21.8 11.8	48 179 66 44	14.2 53.1 19.6 13.1	54 229 102 47	12.5 53 23.6 10.9	0,458^b^
Frequency attending Specialist Care(Only those answering “yes” to healthcare question answer)	Never Once Twice 3 or more times		235 312 112 110	30.6 40.6 14.6 14.3	112 130 48 47	33.2 38.6 14.2 13.9	123 182 64 63	28.5 42.1 14.8 14.6	0,813^b^
Frequency attending Hospital Care(Only those answering “yes” to healthcare question answer)	Never Once Twice 3 or more times		587 90 37 55	76.3 11.7 4.8 7.2	242 44 21 30	71.8 13.1 6.2 8.9	345 46 16 25	79.9 10.6 3.7 5.8	0,054^b^
Frequency attending Emergency Dept.(Only those answering “yes” to healthcare question answer)	Never Once Twice 3 or more times		423 215 65 66	55 28 8.5 8.6	185 96 20 36	54.9 28.5 5.9 10.7	238 119 45 30	55.1 27.5 10.4 6.9	0,054^b^
Unit/speciality you were attended in. (Only those answering “yes” to previous question answer)	Unit of Pain	Yes No	185 584	24.1 75.9	84 253	24.9 75.1	101 331	23.4 76.6	0,619^b^
	Rehabilitation	Yes No	212 557	27.6 72.4	101 236	30 70	111 321	25.7 74.3	0,188^b^
	Traumatology	Yes No	367 402	47.7 52.3	172 165	51 49	195 237	45.1 54.9	0,104^b^
	Rheumatology	Yes No	173 596	22.5 77.5	70 267	20.8 79.2	103 329	23.8 76.2	0,312^b^
	Neurology	Yes No	175 594	22.8 77.2	74 263	22 78	101 331	23.4 76.6	0,641^b^
	Internal medicine	Yes No	240 529	31.2 68.8	102 235	30.3 69.7	138 294	31.9 68.1	0,618^b^
	Oncology	Yes No	41 728	5.3 94.7	21 316	6.2 93.8	20 412	4.6 95.4	0,327^b^
	Neurosurgery	Yes No	91 678	11.8 88.2	50 287	14.8 85.2	41 391	9.5 90.5	0,023^b^
	Physiotherapy	Yes No	282 487	36.7 63.3	134 203	39.8 60.2	148 284	34.3 65.7	0,116^b^
Time waiting for first appointment Unit of Pain (days)	Mean (SD) Median (IR)		313.95 (291.5) 365 (305)		342.22 (294.75) 365 (275)		291.46 (288.49) 365 (320)		0.207^a^

*SD *Standard deviation; IR: Interquartile range

^a^Mann-Whitney U

^b^Chi-squared

Statistical differences were also found in the characteristics of pain between men are women. The most common diagnoses among the women were related to musculoskeletal disorders such as arthrosis (35.6%), osteoporosis (35.5%), neck pain (49%) and migraine (37.4%). In the case of the men, pain related to surgical intervention (17.7%) was the most common. The location of pain also differs depending on sex/gender: women had more widespread pain (16.2%) and pain in the limbs and/or joints (33.1%). However, the back was the part of the body most affected among the men (33.4%). Additionally, women suffered more sleep problems because of their pain (66.7% vs. 62%) and severe and unbearable intensity of pain with more frequency (65% vs. 57.8%) (Table 2).

Regarding the limitations in daily activities, women were more limited (“yes, a lot, somewhat”) than men in feeding themselves (29.5% vs. 21.2%). However, men were more limited in dressing and undressing (49.3% vs. 45.5%).

The use of the health system was very similar between sexes/genders. The only difference was that men were attended more frequently in the neurosurgery department than women (50.7 vs. 9.5%).

### Factors associated to mental component summary of the SF-12

The multivariate analysis of the variables associated with the physical component (PCS-12) showed several similarities between sex/gender. The older people (women B= -0.196; men B=-0.151), and those with a higher intensity of pain (women B= -1.557; men B= -1.435), a higher level of depression (women B= -0.404; men B= -0.322), and those who needed to ask for sick leave due to their pain (women B= -3.816; men= -3.439) obtained lower score in the physical component of SF-12 (PCS-12). Regarding the employment status, similarities were also found. Those reporting disability (women B= -10.541 men B= -7,544) and being retired (women B=-2.454; men B= -1.937) scored lower on the PCS-12. Additionally, the women who were unemployed (B= -3.394) obtained lower score on the PCS-12 than those working (Table 3).

**Table 3:** Factors associated to Physical HRQL in women and men

Variables	MODEL 1: Women Physical HRQL (N = 967)			MODEL 2: Men Physical HRQL (N =692)		
	Beta (SE)	CI 95%	p-value	Beta (SE)	CI 95%	p-value
Constant	47.705	(42.473;52.936)	<0.001	37.010 (3.785)	(29.578;44.443)	<0.001
-Age	-0.196 (0.025)	(-0.246;-0.147)	<0.001	-0.151 (0.031)	(-0.212;-0.091)	<0.001
-Intensity of pain	-1.557 (0.177)	(-1.904;-1.210)	<0.001	-1.435 (0.201)	(-1.830;-1.040)	<0.001
¿Have you taken sick leave in the last year due to your pain?						
-Yes	-3.816 (0.655)	(-5.102;-2.530)	<0.001	-3.439 (0.742)	(-4.897;-1.981)	<0.001
-No *						
HADS Depression	-0.404 (0.063)	(-0.528;-0.279)	<0.001	-0.322 (0.078)	(-0.475;0.168)	<0.001
Employment status						
-Unemployed	-3.394 (0.866)	(-5.093;-1.696)	<0.001	-0.576 (1.085)	(-2.706;1.555)	0.596
-Retired	-2.454 (0.980)	(-4.378;-0.530)	0.012	-1.937 (1.015)	(-3.930;-0.055)	0.057
-Disability	-10.541 (1.195)	(-12.886;-8.196)	<0.001	-7.544 (1.431)	(-10.354;-4.733)	<0.001
-Studing			0.572	0.429 (2.910)		0.883
-Housewife/husband	-0.787 (1.392)	(-3.520;-1.945)	0.082	-2.732 (2.842)	(-5.285;6.142)	0.337
-Working*	-1.666 (0.957)	(-3.544;-0.212)			(-8.313;2.848)	
Fibromyalgia:						
-Yes	-4.787 (0.793)	(-6.344;-3.231)	<0.001			
-No *						
Arthrosis:						
-Yes				-2.521 (0.764)	(-4.022;-1.020)	<0.001
-No *						
Diabetic neuropathy and other neuropathies						
-Yes				-3.871 (1.209)	(-6.245 ;-1.497)	<0.001
-No *						
Low Back Pain						
-Yes				-2.199 (0.6663)	(-3.500;-0.898)	<0.001
-No *						
Educational level						
-Primary studies				0.214 (1.653)	(-3.031;3.460)	0.897
-Secondary studies				-0.030 (0.869)	(-1.735;1.676)	0.973
-University studies				2.079 (0.783)	(0.540;3.617)	0.008
-No studies*						

Dependent variable: The PCS-12 of SF-12v2; SE, Standard error; CI, Confidence interval; * Reference category

Model 1: Adjusted R^2^ = 0.376

Model 2: Adjusted R^2^ =0.320

Focusing on the differences in the PCS-12 by sex/gender, the women with a diagnosis of fibromyalgia (B= -4.787) scored lower. Men diagnosed with arthrosis (B= -2.521), neuropathy (B= -3.871) and low back pain (B= -2.199) obtained lower score. On the other hand, the men with university studies obtained higher scores on the PCS-12 (B = 2.079) compared to those with no studies (Table 3).

### Factors associated with the mental component summary of SF-12

The multivariate analysis of the variables associated with the MCS-12 showed also resemblances between sex/gender. Younger people (women B = 0.111; men B = 0.058), those with higher levels of anxiety (women B= -1.067; men B= -0.101) and higher levels of depression (women B= -0.817; men B= -1.004) scored lower on the MCS-12.

Regarding women’s mental-related quality of life, those with a diagnosis of herniated discs (B= -5324) and those with dysmenorrhea/endometriosis (B= -3.975) scored lower on the MCS-12. However, those with secondary studies (B = 1.699) obtained a higher score on the MCS-12 compared to those without formal studies (Table 4).

**Table 4:** Factors associated to Mental HRQL in women and men

Variables	MODEL 1: Women Mental HRQL (N =1068)			MODEL 2: Men Mental HRQL (N =752)		
	Beta (SE)	CI 95%	p-valor	Beta (SE)	CI 95%	p-value
Constant	31.466 (5.740)	(20.204;42.729)	<0.001	47.388 (2.178)	(43.112;51.665)	<0.001
Age	0.111 (0.016)	(0.079;0.143)	<0.001	0.058 (0.020)	(0.019;0.098)	0.004
Anxiety (HADS)	-1.067 (0.082)	(-1.228;0.906)	<0.001	-0.877 (0.101)	(-1.075;-0.680)	<0.001
Depression ( HADS)	-0.817 (0.080)	(-0.973;-0.660)	<0.001	-1.004 (0.101)	(-1.202;-0.806)	<0.001
Educational level						
-Primary studies	0.906 (0.958)	(-0.974;2.786)	0.345			
-Secondary studies	1.699 (0.675)	(0.376;3.023)	0.012			
-University studies	0.493 (0.562)	(-0.610;1.597)	0.381			
-No studies*						
Herniated discs						
-Yes	-5.324 (2.230)	(-9.700;-0.948)	0.017			
-No *						
Dysmenorrhea/Endometriosis						
-Yes	-3.975 (1.800)	(-7.508;-0.443)	0.027			
-No *						
Pain localization						
-Head				-1.055 (1.361)	(-3.726;1.616)	0.438
-Neck				3.785(1.252)	(1.327;6.244)	0.003
-Back				3.108 (1.046)	(1.055;5.160)	0.003
-Joints				4.375 (1.064)	(2.287;6.463)	<0.001
-Chest				1.634 (2.554)	(-3.380;6.648)	0.522
-Abdomen				0.245 (1.789)	(-3.268;3.758)	0.891
-Others				0.188 (2.858)	(-5.422;5.799)	0.948
Generalized *						
Neck pain						
-Yes				-1.142 (0.602)	(-2.324;-0.039)	0.058
-No *						
Social support (Duke-UNC-11)				0.073 (0.029)	0.016;0.129)	0.012

Dependent variable: The MCS-12 of SF-12v2; SE Standard error, *CI* Confidence interval

Model 1: Adjusted R2 = 0.561

Model 2: Adjusted R2 = 0.571

*Reference category

The men whose pain was located in the neck (B = 3.785), back (B = 3.108) and joints (B = 4.375) scored higher on the MCS-12 compared to those with widespread pain. Additionally, men who perceived having social support also scored higher on the MCS-12 (B = 0.073). On the contrary, men who had a diagnosis of neck pain (B= -1.142) had a lower score on the MCS-12 (Table 4).

## Discussion

Based on a survey of the Spanish general population, this study analyses the differences in the characteristics and consequences of pain between women and men with CP and the factors associated with their HRQL.

The main findings obtained reveal that age, intensity of pain, being on a sick leave due to pain, and having depression were factors that affect the physical quality of life in both sexes/genders. These results are consistent with previous research [31] that shows that aging is a significant factor in the development of chronic pain, as a result of the progressive aging of the global population, CP is becoming increasingly prevalent [32]. The associations and relationships among intensity of pain, depression and being on a sick leave with physical health in CP have been widely discussed. Depression is a common comorbidity in pain conditions and comorbidity is one of the major factors influencing work disability [33]. The associations between pain and depression seem to be reciprocal, although there is stronger evidence that pain predisposes to depression than vice versa [34]. Likewise, the greater the intensity of pain, the less likelihood of remission of depression through anti-depressants, possibly increasing the suffering, prolonging sick leaves [35] and further undermining physical health.

The separate analyses for women and men showed different patterns of factors associated with physical quality of life. More specifically, the results showed that having fibromyalgia affects the physical quality of life of women in particular, which makes sense since women are diagnosed with fibromyalgia much more likely than men [36]. Some authors [37] demonstrated that patients with fibromyalgia tend to have worse health status and quality of life when compared to other patients with other chronic diseases. Due to the complexity of the illness, many women have difficulties in describing their symptoms. Health professionals do not always identify the patient’s CP as being fibromyalgia-related or still do not believe in this condition reported by patients [37]. A large number of studies have reported that patients with fibromyalgia encounter stigmatization, including expressions of disbelief as to the validity of the diagnosis itself, being seen as the medicalization of psychosocial problems [38]. Thus, it is usually only diagnosed in the advanced stages, when it has already had an impact on their physical health.

Having arthrosis, diabetic neuropathy and low back pain were the factors related to the worst physical quality of life of men. Previous studies have found a higher frequency of diabetic sensorimotor polyneuropathy in males, despite the evidence of the higher frequency and intensity of pain and other neuropathic symptoms in females. However, females have greater sensitivity to multiple sensory modalities, which might potentially facilitate early detection and treatment of diabetes, and therefore explain the relatively milder nerve injury in females with diabetes [39]. Low back pain is the most prevalent chronic pain (Table 2) and one of the most common reasons for visiting a physician. Facet arthrosis is a common radiographic finding and has been a suggested cause of low back pain, which has a greater prevalence in men at all lumbar levels [40]. Previous studies have shown that evidence of facet arthrosis can be linked to the amount of heavy work done before the age of 20 [41]. This could be related to another of our results that shows that men with university studies, who usually do not perform manual jobs, had better physical health compared to those with no studies.

Regarding mental quality of life, there were factors that affect both sexes/genders such as age, anxiety and depression. In this study, we found that age was associated with both physical and mental health in both sexes/genders. However, being older was worse for physical quality of life, while it was better for mental quality of life. There is an enormous variety in how the elderly attempt to adjust to the challenge of pain [42]. In this vein, it is noticeable that, as other studies show, elderly patients accept pain as part of their aging process [43], and depression is less commonly associated with pain in elderly individuals than in younger subjects. It can be argued that the duration of pain, which is generally longer in older people, allows them to get used to it and make adjustments over an extended time. Social comparison (comparisons between the self and others) is also important because concern about a symptom is less likely when that symptom is common [44].

The women without studies and those with dysmenorrhea/endometriosis and herniated discs were linked with worse mental quality of life. Regarding the level of studies, lower health literacy has been shown [45, 46] to be associated with poorer self-management behaviour, such as worse adherence to medication, and with greater pain intensity and attention and memory problems, issues that have been acknowledged in CP and depression (the extra burden), which could explain the association in our results with mental health.

Concerning endometriosis, it is a chronic health condition, yet it is still all too often considered taboo or not important due to its links with the menstrual cycle [47]. It is characterized by chronically painful menstruation and sex, and pelvic symptoms, pain stigma that contributes to endometriosis stigma and to poorer mental health. Additionally, infertility is a likely contributor to poorer mental health in people with endometriosis, as up to half of endometriosis patients experience it [48]. On the subject of herniated discs, epidemiological studies have determined that this is more common among women and that the incidence depends on the degree of the lumbar burden associated with tasks performed at the patient’s workplace [49]. Additionally, patients with lumbar or cervical disc herniation had a higher prevalence of anxiety disorders [50], and this is more prevalent in females [51].

The men in the study who had a diagnosis of neck pain, or had widespread pain, and lacked social support had a worse mental quality of life. Our results are not surprising since individuals with widespread pain often experience long-lasting pain in multiple body regions, and this pain is associated with other physical symptoms such as fatigue, concentration problems and psychological distress. In addition, associations between widespread pain and social factors have been reported [52]. That is in line with another of our results: how social support serves as a protective factor, suggesting that it reduces psychological distress especially at times of heightened social isolation, which is common among individuals with chronic pain [53]. Research has demonstrated that many people with psychological problems are reluctant to seek help from mental health professionals or communicate with their social sphere. When it comes to help-seeking for a mental health problem, this process is ultimately determined by structural factors. Gender socialization and traditional male values are impediments to help seeking and inhibit propensities to communicate since men are not expected to express their emotions, which can perpetuate low mental health in this population [54].

Some strength and limitations of the present study must be taken into account. First, the sample, given the characteristics of the online survey, was selected from people that volunteered to be part of the register of a market research company, and would appear to be selective and biased. However, some authors [55] have argued that panel providers undertake to manage the demographic compositions of their respondent pools, and try to correct the biases so that panels are demographically similar to national populations. In addition, online panel surveys are widely used by social scientists. Second, while the sample (*n* = 1825) was robust for analysing the PCS, it was underpowered for the MCS. The observed power of 48% (below the 80% benchmark) increases the risk of Type II errors and limits the generalizability of the MCS related findings. These results should therefore be interpreted with caution. Nevertheless, this study was carried out using a large representative Spanish sample and a rigorous selection procedure. Furthermore, the use of validated scales to measure many dimensions ensures that, despite the aforementioned limitation, the information obtained remains highly valid and reliable. Finally, it is necessary to bear in mind that since this is a cross-sectional study, the relationships observed do not allow us to establish causal relationships.

## Conclusions

CP has an impact on the HRQL of both sexes/genders. However, some issues have a different impact on the HRQL of each sex/gender such as fibromyalgia or endometriosis in women; or low back pain or social support in men. Identifying the sources of this difference in pain is a complex matter that requires a bio-psycho-social perspective. Yet this survey aims to contribute to future data collection efforts and ongoing and future applications centred on the care of both men and women suffering from chronic pain.

## Data Availability

The datasets used and/or analysed during the current study are available from the corresponding author on reasonable request.

## Abbreviations

CP: Chronic pain
CATI: Computer-assisted telephone interviewing
CAWI: Computer-assisted web interviewing
HADS: Hospital Anxiety and Depression Scale
HRQL: Health-related quality of life
IASP: International Association for the Study of Pain
MCS: Mental component
PCS: Physical component
SF-12: Short-Form Health Survey version 2
VIF: Variance inflation factor
